# Have General Surgery Practices Decreased During the COVID-19 Pandemic?

**DOI:** 10.7759/cureus.27270

**Published:** 2022-07-26

**Authors:** Halit Batuhan Demir, Ebubekir Korucuk, Almir Miftari, Yigit Turk

**Affiliations:** 1 Department of General Surgery, Ege University, İzmir, TUR

**Keywords:** sars-cov-2, covid-19, count, operation, general surgery, surgery

## Abstract

Background

As the coronavirus disease 2019 (COVID-19) pandemic started, some restrictions were imposed throughout the country. The pandemic caused disruption, reduction, and even a halt in health services worldwide. During this period, the number of procedures performed in surgical clinics decreased due to the interruption of services and the restriction in patient admissions.

Methodology

In this study, we aimed to evaluate the effect of the pandemic on the number of surgeries performed in our clinic during the pre- and post-pandemic period by evaluating the following elective surgeries conducted between September 2018 and September 2021: upper gastrointestinal system, abdominal wall hernia, gallbladder surgeries, and kidney transplantation.

Results

A significant decrease was observed in the number of operations before and after the pandemic in our clinic.

Conclusions

In our opinion, both the Ministry of Health and healthcare institutions should increase the necessary precautions, organize the planning and programming in hospitals, and increase efforts in protecting healthcare workers and patients by increasing surgical practices and ensuring that the healthcare services we provide reach the numbers noted during the pre-pandemic period.

## Introduction

In December 2019, the severe acute respiratory syndrome coronavirus 2 (SARS-CoV-2) was identified through the results obtained by evaluating patients who were referred to the hospital due to upper and lower respiratory tract infections in Wuhan, China. The disease caused by this virus was named coronavirus disease 2019 (COVID-19) [1]. COVID-19 begins with nonspecific symptoms such as fever, cough, diarrhea, headache, and shortness of breath, progresses to severe pneumonia and acute respiratory distress syndrome, and culminates in respiratory failure and by other mechanisms leads to multiorgan failure with high mortality rates [2,3]. The high transmission rates and the severe course of the disease have led to an increase in the number of patients presenting with COVID-19-like symptoms and an increase in the hospitalization of COVID-19 patients with complications in inpatient clinics and intensive care units [4]. With the declaration of a pandemic by the World Health Organization (WHO) on March 11, 2020, due to COVID-19 [5], life changes and regulations in line with pandemic conditions began to be implemented in Turkey. Therefore, the Ministry of Health and hospital administrations developed specific policies to provide health services. Due to the high transmission rates of the disease, increase in the number of patients, and increase in intensive care hospitalizations and mortal cases, the prohibitive practices were extended. In addition, patients who were scheduled for surgery could not be operated on due to restrictions in outpatient clinics, limitations in hospitalizations, delay in diagnostic tests, delay in oncological treatments, reduction in operating rooms, and an inability to diagnose and treat people with chronic diseases. With the declaration of the pandemic in March 2020, multiple meetings were held at the Ege University Medical Faculty Hospital to quickly create working conditions suitable for the pandemic. As a result of the decisions taken, anesthesiologists were re-distributed to COVID-19 intensive care units and elective operations were reduced. As the COVID-19 case numbers continued to increase, elective operations stopped entirely, leaving only oncological and emergency surgeries to be performed. Oncological surgeries were then deferred as the situation became untenable. In the Department of General Surgery, the decisions taken by the Dean’s office were followed, and operations were temporarily suspended at certain times. In this study, we aimed to evaluate the effects of the pandemic on the number of surgeries performed in our clinic by evaluating elective upper gastrointestinal system surgery, anterior abdominal wall hernia surgery, gallbladder surgery, and kidney transplantation before and after the pandemic.

## Materials and methods

Patients who underwent elective surgeries such as upper gastrointestinal system surgery, anterior abdominal wall hernia surgery, gallbladder surgery, and kidney transplantation were included in our study anonymously. Emergency cases were excluded. Patients who underwent these surgeries between September 2018 and September 2021 were evaluated retrospectively using Ege University Medical Faculty Hospital Electronic Patient File System. The surgeries performed on patients were divided into the following two groups: pre- and post-pandemic. March 11, 2020, the day when the WHO declared a pandemic, was used as a division point for the two groups, and an equal time sequence was evaluated for each group. Data were collected from patients who underwent surgery for esophageal cancer, gastric cancer, peritoneal carcinomatosis, pancreatic cancer, gastrointestinal stromal tumors (GIST), morbid obesity, cholelithiasis, hiatus hernia, umbilical hernia, inguinal hernia, femoral hernia, incisional hernia, epigastric hernia, chronic renal failure, donor for renal transplantation, achalasia, hypersplenism, and splenomegaly. The operation groups named A, B, and C were determined according to the interventional procedures list published by the Ministry of Health of the Republic of Turkey. In this list, the operation groups are determined according to the complexity and difficulty of the procedures, as well as the level of experience and knowledge that the operator should have. Accordingly, group A operations can be defined as large, difficult, and requiring high experience operations (gastrectomy, esophagectomy), group B operations can be defined as less difficult and requiring experience operations (cholecystectomy, laparoscopic hernia repair), and group C operations can be defined as operations requiring moderate experience (umbilical hernia repair) [6]. In the post-pandemic group, all patients who underwent operation were tested for COVID-19 and none tested positive. At the same time, the COVID-19 polymerase chain reaction (PCR) test was repeated for patients with COVID-19 symptoms during postoperative hospitalization, and no positivity was detected in any of the patients. Data were analyzed using the SPSS program (IBM Corp., Armonk, NY, USA). Normally distributed data were analyzed using the Kolmogorov-Smirnov and Shapiro-Wilk tests, and non-normally distributed data were analyzed using the Mann-Whitney U test. Pearson chi-square test was used to compare the data. A p-value of <0.05 was accepted as statistical significance in all analyses.

## Results

**Figure 1:**
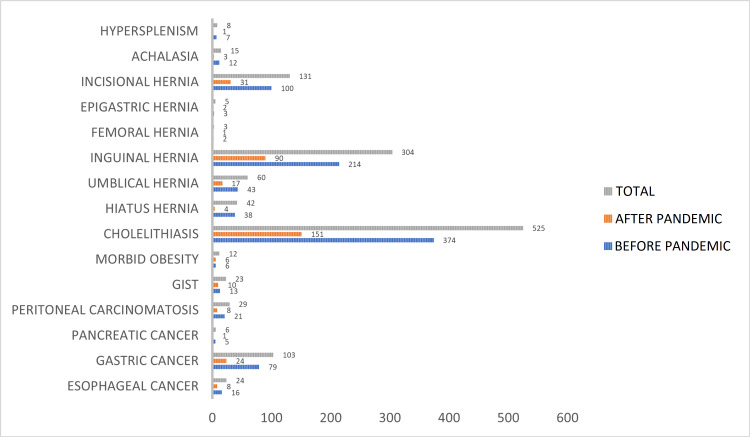
Comparison of preoperative diagnosis: Before and after the pandemic. GIST: gastrointestinal stromal tumor

A total of 1,420 patients were operated on electively in our clinic between September 2018 and September 2021, with the described indications included in the study. These patients were divided into two groups, namely, pre-pandemic and post-pandemic, and March 11, 2020, was used as a division point for the two groups. There were no canceled elective operations. There were 1,033 (72%) patients in the pre-pandemic group and 387 (27.2%) patients in the post-pandemic group. A significant difference was found between these two groups (p < 0.05). Of the operated patients, 782 (55.1%) were male, and 638 (44.9%) were female. In the pre-pandemic group, 560 (54.2%) patients were male, and 473 (45.8%) were female. In the post-pandemic group, 222 (57.3%) were male, and 165 (42.7%) were female. The mean age of the operated patients was 54.57. The mean age of males was 55.13, and the mean age of females was 53.88. In the pre-pandemic group, the mean age of all patients was 54.76, with a mean age of 55.27 for males and 54.16 for female patients. In the post-pandemic group, the mean age of all patients was 54.07, with a mean age of 54.82 for males and 53.07 for female patients. The demographic characteristics of the patients were similar in both groups. In total, 1,242 (87.4%) operations were performed for benign and 178 (12.6%) for malignant diseases. In the pre-pandemic and post-pandemic groups, 134 (12.9%) and 44 (11.3%) patients were operated on for malignant diseases, respectively (p > 0.05). In the pre-pandemic group, 899 (87.1%) patients, and in the post-pandemic group, 343 (88.7%) patients were operated on for benign diseases (p > 0.05) In our study, the most common preoperative diagnosis in the operated patients was cholelithiasis in both groups. Incisional hernia and gastric cancer were the most common in both groups, respectively (Figure 1).

In total, 372 (26.2%) operations were group A operations, 757 (53.3%) were group B operations, and 291 (20.5%) were group C operations. In the pre-pandemic group, 287 (27.8%) operations were in group A, 531 (51.4%) were in group B, and 215 (20.8%) were in group C. In the post-pandemic group, 85 (21.9%) operations were in group A, 226 (58.4%) were in group B, and 76 (19.7%) were in group C. A significant difference was found regarding the surgical groups of the operations performed in the pre-pandemic and post-pandemic groups (p = 0.041). Overall, 600 (42.2%) operations were performed with the open technique and 820 (57.8%) with the laparoscopic technique. In the pre-pandemic group, 452 (43.8%) operations were performed with the open and 581 (56.2%) were performed with the laparoscopic technique. In the post-pandemic group, 148 (38.2%) operations were performed with the open and 239 (61.8%) with the laparoscopic technique (p = 0.064). Minimal significant differences were observed in the pre- and post-pandemic groups regarding operation technique (Table 1).

**Table 1:** Comparison of demographic and operation data: Before and after the pandemic.

Parameters	Pre-pandemic	Post-pandemic	Total
Count of surgeries, n	1,033	387	1,420
Gender, n (%)			
Male	560 (54.2%)	222 (57.3%)	782 (55.1%)
Female	473 (45.8%)	165 (42.7%)	638 (44.9%)
Average age (years)	54.76	54.07	54.57
Male	55.27	54.82	55.13
Female	54.16	53.07	53.88
Preoperative indication, n (%)			
Malignant	134 (12.9%)	44 (11.3%)	178 (12.6%)
Benign	899 (87.1%)	343 (88.7%)	1,242 (87.4%)
Surgical technique, n (%)			
Open	452 (43.8%)	148 (38.2%)	600 (42.2%)
Laparoscopic	581 (56.2%)	239 (61.8%)	820 (57.8%)

Of the surgeries performed with the open technique in the pre-pandemic group, 180 (39.8%) were group A operations, 58 (12.8%) were group B operations, and 214 (47.4%) were group C operations. In total, 107 (18.4%) of the surgeries performed with the laparoscopic technique were group A operations, 473 (81.4%) were group B operations, and one (0.2%) was group C operations. Of the surgeries performed with the open technique in the post-pandemic group, 52 (35.1%) were group A operations, 21 (14.2%) were group B operations, and 75 (50.7%) were group C operations. Of the surgeries performed with the laparoscopic technique, 33 (13.8%) were group A operations, 205 (85.8%) were group B operations, and one (0.4%) was a group C operation (Table 2).

**Table 2:** Comparison of operation groups according to technique: Before and after the pandemic.

	Pre-pandemic		Post-pandemic	
Operation groups	Open	Laparoscopic	Open	Laparoscopic
A Group, n (%)	180 (39.8%)	107 (18.4%)	52 (35.1%)	33 (13.8%)
B Group, n (%)	58 (12.8%)	473 (81.4%)	21 (14.2%)	205 (85.8%)
C Group, n (%)	214 (47.4%)	1 (0.2%)	75 (50.7%)	1 (0.4%)

In the pre-pandemic group, 123 (91.8%) of group A and 11 (8.2%) of group B operations were performed. In contrast, no group C operations were performed due to malignant disease. In the post-pandemic group, 39 (88.6%) group A operations, four (9.1%) group B operations, and one (2.3%) group C operation were performed due to malignant disease. In the pre-pandemic group, 164 (18.2%) group A operations, 520 (57.9%) group B operations, and 215 (23.9%) group C operations were performed due to benign disease. In the post-pandemic group, 46 (13.4%) group A operations, 222 (64.7%) group B operations, and 75 (21.9%) group C operations were performed due to benign disease (Table 3).

**Table 3:** Comparison of operation groups according to preoperative indication: Before and after the pandemic.

	Pre-pandemic		Post-pandemic	
Operation groups	Malignant	Benign	Malignant	Benign
A Group, n (%)	123 (91.8%)	164 (18.2%)	39 (88.6%)	46 (13.4%)
B Group, n (%)	11 (8.2%)	520 (57.9%)	4 (9.1%)	222 (64.7%)
C Group, n (%)	0 (0%)	215 (23.9%)	1 (2.3%)	75 (21.9%)

## Discussion

After the WHO declared the pandemic, arrangements were made in hospitals, including private hospitals, by pandemic conditions and decisions of the Ministry of Health. During this process, an initial restriction on the number of patients was applied for outpatient clinics to operating rooms, followed by total closure. Thus, all elective surgeries were postponed except for semi-urgent oncological cases. With the decision of the Dean and Chief Physician Office of the Ege University Faculty of Medicine Hospital, the total lockdown in the General Surgery Operation room started on March 15, 2020, and continued until June 02, 2020. During this period, urgent oncological cases such as cancer patients who received neoadjuvant treatment and patients with increasingly deteriorating status were operated on. Emergency cases were also operated during this period, but they were not included in our study. With the developments in the prevention and treatment methods of COVID-19, health institutions became more organized over time, and public awareness of the pandemic got more robust; the practices of closing the operating theaters came to an end. The operating room of our clinic started to operate at half capacity with the end of total lockdown. According to the data we obtained, significantly fewer operations were performed in the post-pandemic period than in the pre-pandemic period. We think that the three to four months of full closure and working at half capacity is the main reason for this severe decrease in the number of surgeries performed. Moreover, the decrease in outpatient numbers, the decrease in the inpatient capacity, and the prohibitions in social life may have caused the decrease in surgeries. Due to the decrease in the number of operations, many studies have reported that this causes a delay in the treatment of patients with malignant diseases and those who need curative surgical treatment and consequently causes severe morbidity and mortality [7,8]. In our study, there was a decrease in the number of surgeries for malignant diseases due to the decrease in the number of surgeries. However, there was no significant difference in the ratio of the number of surgeries with malignant diseases between pre-pandemic and post-pandemic groups. Considering that urgent oncological cases were prioritized in the operation plans, and ensuring that the treatment of patients with malignancy was not delayed during the half-capacity working period, an increase in the number of surgeries performed with malignant indication should have been observed in the post-pandemic period. However, a decrease in outpatient visits, a decrease in the inpatient capacity of the wards, and a decrease in the meetings held for malignant diseases requiring multi-disciplinary evaluation may have played a role in the absence of this increase. At the beginning of the pandemic, it was hypothesized that laparoscopic surgery, in which airflow played a vital role, increases the virus spread due to transmission through droplets and inhalation [9]. However, studies have shown no significant difference between open and laparoscopic surgery in terms of spread and transmission of the virus [10]. In our study, we noticed that laparoscopic surgery was performed more often than open surgery both in the pre-pandemic and post-pandemic groups. Although the number of laparoscopic surgeries increased in the post-pandemic period compared to the pre-pandemic period, no significant difference was observed. The fact that laparoscopic surgery was preferred more frequently in group B and the significant increase in the number of group B surgeries performed in the post-pandemic group may have caused the rates of laparoscopic surgery. A study conducted with 138 patients diagnosed with cancer in China found that 41% of the patients were infected with the SARS-CoV-2 virus after hospitalization. Therefore, it was argued that hospitals are one of the riskiest places for transmission of COVID-19 [11]. With the start of the pandemic, in our clinic, attention was paid to preventing SARS-CoV-2 transmission as the Ministry of Health recommended and was regularly inspected. In this context, unnecessary materials that carry the risk of contamination were not kept in operating rooms, and personnel who were not active when the risk of inhalation-related contamination, such as intubation and insufflation increased, were kept out the room. Additional personnel and technicians were assigned to supervise material transport as the material inside and outside the operation room and decrease transmission. Thus, the risk of SARS-CoV-2 contamination of healthcare workers and patients was reduced as much as possible. COVID-19 PCR test positivity was not observed in any of the 387 patients in our study who were operated on in the post-pandemic group.

In our study, only operations performed due to upper gastrointestinal diseases and kidney transplantation were included. Considering other general surgery applications such as colorectal, hepatobiliary, and breast operations could have provided more data and more objective results regarding general surgery. Again, conducting a single-center study was one of the limitations of the study.

## Conclusions

The COVID-19 pandemic has caused disruption, reduction, and even halt in health services worldwide. During this period, the admission rate in surgical clinics decreased due to both the interruption of services and the restriction of patient visits. Although we have improved disease prevention and fight against the disease, we have not yet been able to achieve complete isolation and return to routine life and provision of health services just like in the pre-pandemic era. In our opinion, both the Ministry of Health and health institutions should increase the necessary precautions, organize the planning and programming in hospitals, and increase efforts in protecting health workers and patients to increase surgical practices and ensure that the health services we provide reach the numbers noted in the pre-pandemic period.
